# Not so FAST: pre-hospital posterior circulation stroke

**DOI:** 10.29045/14784726.2022.06.7.1.24

**Published:** 2022-06-01

**Authors:** Shane Devlin

**Affiliations:** National Ambulance Service, Ireland

**Keywords:** fast negative, paramedic, posterior circulation stroke

## Abstract

**Introduction:**

Posterior circulation strokes account for 20% of ischaemic strokes, but may present differently to their anterior circulation counterparts. Patients may not exhibit unilateral facial weakness, speech disturbances and unilateral limb weakness, but instead present with more vague symptoms of sudden headache, dizziness, loss of balance and visual problems. This case describes a patient displaying signs and symptoms of a posterior stroke, but who eluded the FAST (face, arm, speech, time) test.

**Case presentation:**

An ambulance was called for a 60-year-old man who had a sudden onset of generalised weakness, diaphoresis and one episode of emesis at home in rural Ireland. He had a history of hypertension, hypercholesterolaemia, angina and a coronary stent placed 4 months previously. Cardiac, respiratory, abdominal, urinary and gastrointestinal exams were unremarkable. Vital signs and 12-lead electrocardiogram were normal. He was FAST negative on exam. Due to persistent dizziness, further neurological exams were carried out, showing a left visual field neglect, new nystagmus, left-sided dysmetria on finger-to-nose and heel-to-shin tests and he was unable to walk unassisted upon standing. A posterior circulation stroke was suspected, and the nearest stroke unit was pre-alerted en route. A rapid assessment and computed tomography took place at hospital, with timely thrombolysis with tissue plasminogen activator. The patient subsequently had a full neurological recovery.

**Conclusion:**

This case describes a patient displaying signs and symptoms of a posterior circulation stroke albeit being FAST negative on exam. There is potential here to improve our recognition of posterior stroke in the pre-hospital field by including additional neurological exams to the FAST test. Use of ‘BEFAST’ (balance, eyes, face, arm, speech, time), the finger-to-nose test, and the ‘5 Ds’ and ‘DANISH’ mnemonics may help increase recognition of these subtle presentations.

## Introduction

There are 130 billion neurons in the human brain. During a stroke, 1.9 million of these neurons die per minute. Each minute due to ischemia, the brain will age over 3 weeks, for every hour, over 3.5 years, and by the end of the stroke, the brain will have aged 36 years (Saver, 2006). As up to 70% of strokes are first encountered by the emergency medical services (EMS) (Jones et al., 2021), the pre-hospital phase of stroke management is crucial, with studies showing that rapid pre-hospital treatment, transport and hospital notification reduce door-to-computed tomography (CT) and thrombolysis times and improve outcomes (Bae et al., 2010; McKinney et al., 2013; Oostema et al., 2014).

Traditional paramedic teaching encourages using the ‘FAST’ (face, arm, speech, time) test, to assess for facial weakness, arm weakness and speech disturbances (Brown et al., 2019; Pre-Hospital Emergency Care Council, 2018). This test is sensitive at detecting strokes occurring in the anterior circulation, but is less accurate at detecting strokes in the posterior circulation, which account for around 20% of ischaemic strokes. The posterior circulation supplies the cerebellum, brainstem and occipital cortex and so strokes in this part of the brain can present with vague, non-specific signs such as dizziness, visual disturbances, loss of balance and co-ordination, headache and nystagmus (Merwick & Werring, 2014). These patients may be FAST negative on exam. This has the potential to delay diagnosis in the pre-hospital environment and therefore adversely affect patient outcomes and recovery. The purpose of this case report is to create an awareness of posterior circulation strokes and improve their identification in the pre-hospital phase of care.

## Case presentation

An ambulance was called for a 60-year-old man who had a sudden onset of generalised weakness, diaphoresis and one episode of emesis at home. He had a history of hypertension, hypercholesterolaemia, angina and a coronary stent placed 4 months previously. On arrival he appeared well and reported no pain. Vital signs were unremarkable. Lung sounds were clear, and a 12-lead electrocardiogram showed normal sinus rhythm with no acute abnormalities. A FAST test proved negative. Despite these tests proving normal, the patient stated he was still very dizzy. Due to this, further tests examining vision, co-ordination and balance were carried out. Visual field tests showed left visual field neglect, vertical and horizontal nystagmus, finger-to-nose and heel-to-shin tests showed left-sided dysmetria and the patient had an unsteady gait upon standing and could not walk unassisted.

### Management

A posterior circulation stroke was suspected. As local hospital protocols suggest an 18-gauge cannula be inserted for CT contrast, one was placed on-scene rather than en route to hospital, due to the potential difficulty of placing one en route on bad rural roads. The receiving hospital was pre-alerted, requesting the stroke team. The 30-minute journey was uneventful. Upon arrival, a rapid assessment took place and the patient was taken to CT immediately. A posterior stroke was diagnosed, and the patient was thrombolysed with tissue plasminogen activator.

### Follow-up and outcome

On 4-week follow-up, the patient was at home enjoying life as normal with no neurological deficits from the event.

## Discussion

A recent systematic review showed that between 2% and 52% of strokes transported by EMS are not recognised (Jones et al., 2021). Twenty-five per cent were FAST negative and the most common missed symptoms were speech problems and posterior circulation symptoms (Jones et al., 2021). Stroke is less likely to be suspected by EMS in these patients, and therefore the stroke pathway is not activated. Some studies even show that functional outcomes for posterior stroke patients are worse than their anterior counterparts (Kim et al., 2017; Sommer et al., 2018), while another study found that, if treated with thrombolysis and/or thrombectomy, posterior circulation stroke patients had a better functional outcome at 3 months (Handelsmann et al., 2021).

This is an important point – those who were recognised and treated did well. The problem is not with the therapy, it is with *getting these patients to the therapy*. Delayed recognition means delayed or even no treatment, leading to poorer functional outcomes for these patients.

A systematic review of the accuracy of pre-hospital stroke tools showed that up to 30% of strokes are missed by pre-hospital clinicians in the field (Brandler et al., 2014). Most pre-hospital stroke tools are only designed to detect strokes affecting the anterior circulation, mainly occurring in the middle cerebral artery. While these are the most common presentations of stroke, those affecting the posterior circulation are more likely to be missed as they can present with vague non-specific symptoms not commonly picked up by pre-hospital stroke tools (Brandler et al., 2015; Kothari et al., 1999).

One New York study published in 2015 found that New York City Fire Department emergency medical technicians and paramedics missed 37.6% of all strokes in a one-year period. Providers used the Cincinnati Prehospital Stroke Scale / FAST test as per local protocol, and were more likely to miss a stroke with vague and non-specific symptoms such as altered mental status, general weakness, headache, nausea and dizziness. This study highlighted that it may be appropriate to use tests applicable to other vascular beds of the brain by testing vision, eye movement and cerebellar signs to recognise a potential posterior circulation stroke (Brandler et al., 2015). A similar study in Kent, Michigan, showed that the most common impressions among pre-hospital missed strokes included general weakness, altered mental status and dizziness (Oostema et al., 2015). Posterior circulation stroke is associated with prolonged time to CT and delayed thrombolysis on presentation to the emergency department (Sarraj et al., 2013). This is echoed in the pre-hospital environment as well, where an Austrian cohort study showed that approximately 100 people per year with posterior circulation strokes eligible for treatment were lost due to pre-hospital delays (Sommer et al., 2017).

### Additional examinations to detect posterior circulation stroke

This case report highlights some of the limitations of the FAST exam and should encourage pre-hospital clinicians to undertake further neurological exams to assess for possible posterior strokes. This may increase pre-hospital recognition and reduce the timeframe to imaging and treatment.

#### BEFAST

The addition of ‘balance’ and ‘eyes’ – sudden change in gait and/or visual symptoms to the FAST test (BEFAST) has been shown to reduce the missed stroke rate from the normal FAST test from 14% to 4.4% (Aroor et al., 2017). A recent systematic review and meta-analysis comparing FAST to BEFAST showed that BEFAST had a higher diagnostic value than FAST and may have an important role to play going forward in detecting posterior circulation strokes (Chen et al., 2021).

#### The finger-to-nose test

This is a test of upper limb co-ordination. Keep the patient’s head straight and place one finger in front of them. Ask the patient to touch their nose with their index finger, then touch your finger. Repeat with the other hand. Intention tremor exists when the finger and/or arm begins to tremor as the finger almost touches your finger. Dysmetria is a complete lack of co-ordination, shown by missing the target completely.

A recent study showed that training paramedics in the finger-to-nose test alone increased recognition of posterior strokes by 28%. This also decreased the door-to-CT time by 21 minutes. Although a small study, this is a promising finding that even small additions to the FAST test may detect more posterior strokes and enhance care for these patients (Oostema et al., 2019).

#### The 5 Ds

The ‘5 Ds’ of posterior stroke (Brown, 2015) focus mainly on patient symptoms, and although helpful to recall, the paramedic must remember that many other illnesses can cause these symptoms, and not just posterior stroke:

- Dizziness
- Diplopia (double vision)
- Dysarthria (slurred speech)
- Dysphagia (difficulty swallowing)
- Dystaxia (abnormal gait, balance, motor movements)

#### DANISH

Instead of doing a full neurological exam, ‘DANISH’ (Bargiela, 2021; Gudlavalleti & Tenny, 2021) highlights the main points of the exam when looking for posterior stroke signs, and may assist the paramedic with on-scene assessment, particularly if the patient is FAST negative:

- **Dysdiadochokinesia:** This is when a patient is unable to perform rapid alternating movements. Ask the patient to place the palm of their right hand on the palm of their left hand, like they are about to clap. Now ask the patient to turn the right hand over and touch the left palm with the back of the hand. Repeat this as fast as they can. Now repeat with the other hand. If they are unable to do this, it may be a sign of cerebellar ataxia. (Note: examining intention tremor, dysmetria and dysdiadochokinesia may be difficult in the older person for a variety of reasons. Try something else to assess their fine motor skills, like asking them to button their shirt, open a biscuit tin, lifting and drinking a glass of water; something they are normally able to do but may be unable to do today.)
- **Ataxia:** Ataxia consists of abnormalities in gait, movement and posture. Is the patient able to walk as normal, or are they staggering or veering to one side?
- **Nystagmus:** Involuntary, repetitive eye movements. Ask the patient to look straight ahead, and ask them to follow your finger as you make a ‘H’ shape in the four ‘corners’ of vision. Eyes should follow your finger smoothly; the presence of ‘jerky’ movement indicates nystagmus. Note the direction.
- **Intention tremor:** This is examined with the finger-to-nose test.
- **Slurred, staccato speech:** Speech may be slurred, as though the patient is drunk, or broken down into separate phrases or syllables (staccato speech). Ask the patient/family what is normal for them.
- **Hypotonia, heel-to-shin test:**
  - **Hypotonia** – assessment of tone in the upper limbs. Support the patient’s arm by the elbow and wrist and ask them to relax and let you control the movement of the arm. Note any abnormalities in tone (hypotonia/hypertonia). This, however, is subjective, so do not put too much emphasis on this clinical finding pre-hospital.
  - **Heel-to-shin** – lower limb co-ordination. Assess for limb weakness first. Ask the patient to put their right heel on their left shin and run it down their leg. Repeat with the opposite leg. Dysmetria and intention tremor may be seen as similar to the upper limb exam.

## Conclusion

This case report describes a patient suffering from a posterior circulation stroke but who was FAST negative on exam. Persistent dizziness of unknown cause triggered the application of additional neurological exams, which increased the suspicion for posterior circulation stroke. A pre-hospital stroke alert was activated, and the patient was treated successfully by the receiving stroke team, subsequently making a complete neurological recovery. As these patients may be FAST negative on exam, this article suggests the use of some additional exams such as BEFAST, the finger-to-nose test, the 5 Ds and DANISH in patients with acute neurological symptoms. This may help paramedics recognise posterior circulation stroke in the field, and encourage transport to the nearest stroke unit for imaging and treatment. As this is a relatively new area of research for paramedicine, more studies are needed to help guide pre-hospital recognition and stroke team activation for posterior circulation strokes.

## Acknowledgements

I would like to thank the patient and his family for kindly giving their permission to write this case report.

## Author contributions

SD acts as the guarantor for this article.

## Conflict of interest

None declared.

## Ethics

Verbal and written consent were gained from patient and family.

## Funding

None.
